# Chronic Extrahepatic Portal Vein Occlusion in a Young Patient

**DOI:** 10.7759/cureus.40806

**Published:** 2023-06-22

**Authors:** H. S Kiran, Rohan Karkra, V. N Laghima, C. R Venkatesh

**Affiliations:** 1 Department of General Medicine, JSS Medical College and Hospital, JSS Academy of Higher Education & Research (JSSAHER), Mysuru, IND

**Keywords:** non cirrhotic portal hypertension, splenomegaly, abdominal pain, vomiting, protein c and protein s deficiencies, extrahepatic portal vein obstruction (ehpvo)

## Abstract

Extrahepatic portal vein obstruction (EHPVO) is a rare condition characterized by the occlusion or narrowing of the portal vein outside the liver. We present a case report of a patient with EHPVO secondary to combined protein C and S deficiency and pancytopenia secondary to hypersplenism, highlighting the clinical presentation, diagnostic challenges, and management strategies. Early recognition of this condition and prompt initiation of appropriate treatment can prevent life-threatening complications such as variceal bleeding and portal hypertension. This case underscores the need for a high index of suspicion for inherited thrombophilias in patients presenting with portal vein thrombosis, particularly in the absence of traditional risk factors.

## Introduction

The portal vein, accounting for 70% of the circulation reaching the liver is formed by the merging of the splenic vein and superior mesenteric vein. An obstruction can develop in its flow, either within the hepatic anatomy or extrahepatically. Extrahepatic portal vein obstruction (EHPVO) is a vascular disorder affecting the extra-hepatic portal circulation and is characterized by obstruction to the flow of blood, which can be acute or chronic. It is an important cause of noncirrhotic portal hypertension. The causes for EHPVO can vary, but the most common causes are infection, sepsis, pregnancy, oral contraceptive use, protein C and S deficiency, factor V Leiden mutation, antithrombin III deficiency, infiltration by malignancy, etc. [1].

The exact incidence of EHPVO is not well known. In India, the estimated prevalence ranges from 0.84 to 13.6 per 100,000 population [2]. The disorder predominantly affects young individuals, with a peak incidence between 10 and 30 years of age [3]. Male preponderance has been observed in various studies, with a male-to-female ratio of approximately 2:1 [4]. Additionally, there is a familial clustering of EHPVO cases, suggesting a genetic predisposition [5].

EHPVO presents with a wide range of clinical manifestations, making early diagnosis challenging. Many patients remain asymptomatic until the development of complications. Common symptoms include splenomegaly, recurrent variceal bleeding, abdominal pain, and ascites [6]. Splenomegaly, resulting in hypersplenism, is a key clinical finding observed in the majority of patients with EHPVO [7]. Gastrointestinal bleeding due to oesophageal varices is a significant complication, occurring in up to 80% of cases [8]. EHPVO can lead to various complications, some of which can be life-threatening. Recurrent variceal bleeding is the most common and potentially fatal complication [9]. Other complications include hypersplenism-related cytopenias, portal biliopathy (biliary abnormalities due to portal cavernoma compression), and portal vein thrombosis [10]. Hepatic encephalopathy, though rare, can also occur in advanced stages of the disease [11].

The management of EHPVO involves a multidisciplinary approach aiming to alleviate symptoms, prevent complications, and improve the overall quality of life. Long-term anticoagulation is indicated to prevent thrombosis. Nonselective beta-blockers (e.g., propranolol) and endoscopic band ligation (EBL) are the mainstays for primary prophylaxis of variceal bleeding [12]. In patients with acute variceal bleeding, combination therapy with vasoactive drugs, antibiotic prophylaxis, and endoscopic intervention are recommended [13]. Trans-jugular Intrahepatic Portosystemic Shunt (TIPS) placement or surgical shunts may be considered in refractory cases or when endoscopic interventions fail [14]. Liver transplantation remains a definitive treatment option for patients with advanced liver disease or intractable complications [15].

## Case presentation

**Figure 1:**
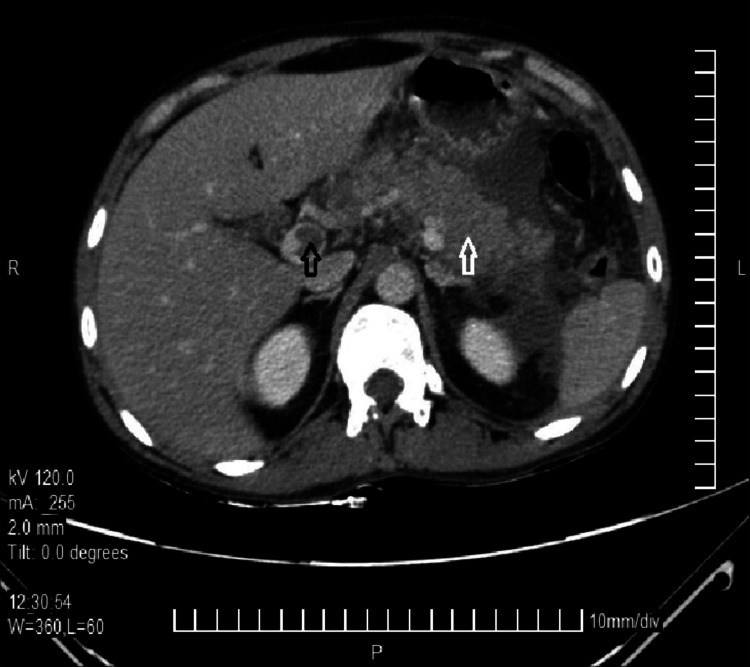
Contrast-enhanced CT scan showing EHPVO Black arrow shows thrombosis of the portal vein and the white arrow shows cavernous transformation of the portal vein and congestion

A 19-year-old male patient with no known comorbidities presented with complaints of sudden onset acute abdominal pain followed by vomiting for one day. The patient described the pain as diffuse, stabbing, and continuous without radiation. The pain did not subside with analgesics. There was associated history of four episodes of vomiting, non-projectile, non-bilious, and containing food particles. There was no history of consumption of outside food, fever, loose stools, or constipation. There was no history of similar complaints in the past or similar complaints in the family. There was no history of substance use or prolonged medication use. On examination, mild tachycardia and pallor were noted. Other vital signs and general physical examination were normal. Per abdominal examination revealed diffuse tenderness, guarding, and mild splenomegaly. The bowel sounds were present and normal. The remaining systemic examination was normal. A provisional diagnosis of intestinal obstruction/superior mesenteric artery (SMA) occlusion was made, and the patient was admitted for further evaluation. Basic laboratory investigations were sent. The patient had a hemoglobin (Hb) of 5.6 g/dL, a total count of 3,400 cells/cumm, and a platelet count of 28,000 cells/cumm. The erythrocyte sedimentation rate (ESR) was 5 mm/hr. A peripheral blood smear revealed microcytic hypochromic anemia with leukopenia and thrombocytopenia. The renal function test (RFT), liver function test (LFT), and blood glucose levels were within normal ranges. Pancreatic enzymes were also within normal limits. An erect abdomen x-ray was ordered and was grossly normal. An ultrasound of the abdomen was ordered which showed signs suggestive of EHPVO. A contrast-enhanced computed tomography (CECT) scan was done which also showed signs suggestive of EHPVO as seen in Figure 1.

An upper gastrointestinal endoscopy showed large esophageal varices, gastric varices, and portal gastropathy. In order to ascertain the cause of EHPVO, other investigations were ordered - Factor V Leiden mutation, Prothrombin mutation, and lupus anticoagulant were negative. JAK 2 mutation tested in view of thrombophilia was also negative. The sickling test and Hb electrophoresis were also negative. Protein C (36% (Normal = 70%-130%)) and Protein S (53% (Normal = 70%-148%)) were lower than normal, hence confirming the thrombophilic etiology.

The patient was diagnosed with EHPVO secondary to combined protein C and S deficiency, pancytopenia secondary to hypersplenism, and portal gastropathy. The patient received symptomatic management and pain relief. Three units of packed red blood cells (PRBCs) were transfused to correct the anemia, with the Hb subsequently rising to 8 g/dL. Variceal ligation was done for the esophageal and gastric varices. The patient was planned to be started on oral anti-coagulants for the EHPVO on follow-up after thrombocytopenia had resolved and was discharged in a hemodynamically stable state.

## Discussion

EHPVO is a condition characterized by the blockage or narrowing of the portal vein outside the liver. This leads to portal hypertension and various clinical manifestations such as abdominal pain, vomiting, hematemesis, etc. EHPVO predominantly affects young individuals in the Indian subcontinent, with a higher incidence in males [2,3]. The exact etiology of EHPVO remains unclear, but several factors have been implicated, including genetic predisposition, prothrombotic states, chronic infections, and environmental factors [1]. Future research should focus on unraveling the genetic and environmental determinants of EHPVO to aid in early identification and prevention strategies. While the etiopathogenesis and symptomatology are similar between EHPVO and intrahepatic venous obstruction, they can be differentiated by the presence of massive splenomegaly, absence of ascites and hepatomegaly, and normal LFTs in EHPVO [10]. Grossly, the liver architecture remains normal in EHPVO.

The clinical features of EHPVO can vary widely, ranging from asymptomatic cases to severe complications such as variceal bleeding, ascites, and hepatic encephalopathy. Splenomegaly is a common finding during physical examinations [6,7]. Prompt recognition of clinical features is crucial for early diagnosis and appropriate management of EHPVO. The clinical manifestations of this condition, in this case, are consistent with EHPVO. Abdominal pain can be a result of portal congestion, organ ischemia- particularly liver, rupture of collateral vessels, or even organomegaly [16]. Vomiting can result from variceal bleeding or pressure from the enlarged spleen on the stomach. Additionally, hepatic dysfunction, often seen in EHPVO, can impair the clearance of toxic compounds and cause vomiting as well [17].

Management strategies for EHPVO aim to alleviate symptoms, prevent complications, and manage underlying portal hypertension. Non-cirrhotic portal fibrosis (NCPF) is a crucial histopathological finding associated with EHPVO, characterized by fibrotic changes in the portal tract and the absence of significant liver parenchymal disease. Medical management includes the long-term (three to six months) use of anticoagulants, such as low molecular weight heparin, warfarin, or directly acting oral anticoagulants (DOAC) such as Dabigatran, Rivaroxaban, etc., to prevent further thrombosis and recanalization of the portal vein [10]. Anticoagulation therapy has shown promising results in improving portal flow and reducing the risk of recurrent thrombosis. However, careful monitoring for bleeding complications is essential, particularly in patients with a history of variceal bleeding.

In cases of symptomatic portal hypertension, endoscopic interventions play a crucial role. EBL and sclerotherapy are commonly employed to manage esophageal varices and prevent variceal bleeding [18]. However, the presence of gastric varices poses a challenge, as they are associated with a higher risk of bleeding and require alternative management strategies. Endoscopic cyanoacrylate glue injection has emerged as an effective treatment modality for gastric varices, offering good hemostasis and reducing the risk of rebleeding [18]. Further research is warranted to evaluate the long-term efficacy and safety of cyanoacrylate glue injection in the management of gastric varices associated with EHPVO.

Surgical interventions, including shunt surgeries and portosystemic decompressive procedures, may be considered in select cases of refractory variceal bleeding or the presence of significant portal cavernoma. Surgical shunts, such as the meso-Rex bypass or portosystemic shunts, aim to divert portal blood flow and reduce portal hypertension [15]. However, these procedures are associated with surgical complications and long-term risks.

## Conclusions

EHPVO is a vascular disorder of the liver, which causes obstruction and cavernous transformation of the portal vein with or without the involvement of the intra-hepatic portal vein, splenic vein, or superior mesenteric vein. EHPVO is one of the most common causes of portal hypertension after cirrhosis. Chronic EHPVO can be asymptomatic for a long and can present acutely as in this case. As non-cirrhotic EHPVO tends to be seen in younger populations, prompt identification and initiation of anticoagulants are important to prevent acute decompensation and complications.

## References

[1] Mantaka A, Augoustaki A, Kouroumalis EA, Samonakis DN (2018). Portal vein thrombosis in cirrhosis: diagnosis, natural history, and therapeutic challenges. Ann Gastroenterol.

[2] Wani ZA, Bhat RA, Bhadoria AS, Maiwall R (2015). Extrahepatic portal vein obstruction and portal vein thrombosis in special situations: need for a new classification. Saudi J Gastroenterol.

[3] Sarin SK, Kapoor D (2002). Non-cirrhotic portal fibrosis: current concepts and management. J Gastroenterol Hepatol.

[4] Goel A, Ramakrishna B, Zachariah U (2019). What makes non-cirrhotic portal hypertension a common disease in India? Analysis for environmental factors. Indian J Med Res.

[5] Caropreso M, Campanile R, Maddaluno S, Veropalumbo C, Piscopo C, Castaldo G, Vajro P (2010). Genetic prothrombotic risk factors in children with extrahepatic portal vein obstruction. J Pediatr Gastroenterol Nutr.

[6] Gioia S, Nardelli S, Pasquale C (2018). Natural history of patients with non cirrhotic portal hypertension: comparison with patients with compensated cirrhosis. Dig Liver Dis.

[7] Gioia S, Nardelli S, Ridola L, Riggio O (2020). Causes and management of non-cirrhotic portal hypertension. Curr Gastroenterol Rep.

[8] Sarin SK, Sollano JD, Chawla YK (2006). Consensus on extra-hepatic portal vein obstruction. Liver Int.

[9] Sarin SK, Kumar A, Chawla YK (2007). Noncirrhotic portal fibrosis/idiopathic portal hypertension: APASL recommendations for diagnosis and treatment. Hepatol Int.

[10] Grus T, Lambert L, Grusová G, Banerjee R, Burgetová A (2017). Budd-Chiari syndrome. Prague Med Rep.

[11] Romero-Gómez M, Montagnese S, Jalan R (2015). Hepatic encephalopathy in patients with acute decompensation of cirrhosis and acute-on-chronic liver failure. J Hepatol.

[12] Tripathi D, Ferguson JW, Kochar N (2009). Randomized controlled trial of carvedilol versus variceal band ligation for the prevention of the first variceal bleed. Hepatology.

[13] de Franchis R (2010). Revising consensus in portal hypertension: report of the Baveno V consensus workshop on methodology of diagnosis and therapy in portal hypertension. J Hepatol.

[14] de Franchis R, Dell'Era A, Primignani M (2008). Diagnosis and monitoring of portal hypertension. Dig Liver Dis.

[15] de Franchis R (2005). Evolving consensus in portal hypertension. Report of the Baveno IV consensus workshop on methodology of diagnosis and therapy in portal hypertension. J Hepatol.

[16] Kumar A, Sharma P, Arora A (2015). Review article: portal vein obstruction--epidemiology, pathogenesis, natural history, prognosis and treatment. Aliment Pharmacol Ther.

[17] Lopes D, Samant H (2023). Hepatic Failure. https://pubmed.ncbi.nlm.nih.gov/30855815/.

[18] Poza Cordon J, Froilan Torres C, Burgos García A, Gea Rodriguez F, Suárez de Parga JM (2012). Endoscopic management of esophageal varices. World J Gastrointest Endosc.

